# Short- and long-term pathologic responses to quartz are induced by nearly free silanols formed during crystal fracturing

**DOI:** 10.1186/s12989-024-00611-8

**Published:** 2024-12-05

**Authors:** Cristina Pavan, Riccardo Leinardi, Anissa Benhida, Saloua Ibouraadaten, Yousof Yakoub, Sybille van den Brule, Dominique Lison, Francesco Turci, François Huaux

**Affiliations:** 1 Louvain Centre for Toxicology and Applied Pharmacology (LTAP), Institute of Experimental and Clinical Research (IREC), Université catholique de Louvain (UCLouvain), Brussels, Belgium; 2 Department of Chemistry, University of Turin, Turin, Italy; 3 “G. Scansetti” Interdepartmental Centre for Studies on Asbestos and Other Toxic Particulates, University of Turin, Turin, Italy

**Keywords:** Quartz, Silica, Respirable crystalline silica, Silanols, Inflammation, Fibrosis, Cancer, Co-carcinogenicity, Autoimmunity

## Abstract

**Background:**

Inhalation of respirable crystalline silica particles, including quartz, is associated with an increased risk of developing pathologies, including persistent lung inflammation, fibrosis, cancer, and systemic autoimmunity. We demonstrated that the nearly free silanols (NFS) generated upon quartz fracturing trigger the early molecular events determining quartz toxicity. Here, we address the involvement of NFS in driving short- and long-term pathogenic responses, including lung inflammation, fibrosis, cancer, and autoimmunity in multiple mouse models.

**Results:**

In vivo pulmonary responses to as-grown NFS-poor quartz (gQ) and fractured NFS-rich quartz (gQ-f) of synthetic origin were compared to two NFS-rich reference quartz dusts (Min-U-Sil 5, mQ-f). Acute and persistent inflammation, as well as fibrosis, were assessed 3 and 60 days, respectively, after administering one dose of particles (2 mg) via oropharyngeal aspiration (o.p.a.) to C57BL/6 mice. The carcinogenic potential was assessed in a co-carcinogenicity study using A/J mice, which were pre-treated with 3-methylcholanthrene (3-MC) and administered four doses of quartz particles (4 × 1 mg, o.p.a.), then sacrificed after 10 months. Autoimmunity was evaluated in autoimmune-prone 129/Sv mice 4 months after particle administration (2 × 1.25 mg, o.p.a). Mice exposed to NFS-rich quartz exhibited a strong acute lung inflammatory response, characterized by pro-inflammatory cytokine release and leukocyte accumulation, which persisted for up to 60 days. No inflammatory effect was observed in mice treated with NFS-poor gQ. Fibrosis onset (i.e., increased levels of pro-fibrotic factors, hydroxyproline, and collagen) was prominent in mice exposed to NFS-rich but not to NFS-poor quartz. Additionally, lung cancer development (tumour numbers) and autoimmune responses (elevated IgG and anti-dsDNA autoantibody levels) were only observed after exposure to NFS-rich quartz.

**Conclusions:**

Collectively, the results indicate that NFS, which occur upon fracturing of quartz particles, play a crucial role in the short- and long-term local and systemic responses to quartz. The assessment of NFS on amorphous or crystalline silica particles may help create a predictive model of silica pathogenicity.

## Background

Respirable crystalline silica (RCS), especially the quartz polymorph, still represents one of the major concerns for respiratory diseases in the occupational field. RCS may cause an impairment of the respiratory function, silicosis, lung cancer, and autoimmune diseases [1, 2]. The main pathological hallmarks related to chronic inhalation of RCS particles consist in persistent lung inflammation, excess deposition of fibrotic tissue, and/or the development of malignant epithelial cells.

Despite the well-known toxic activity of quartz, variable degrees of toxicity were reported, which were ascribed to the heterogeneity of the surface features of each quartz specimen [3, 4]. Depending on the source, mechanical comminution, and physico-chemical treatments, quartz particles showed surfaces that were variably active towards membranolysis models [5, 6], and induction of cell factors related to inflammation and cytotoxicity, including activation of the NACHT, LRR, and PYD domains-containing protein 3 (NLRP3) inflammasome and interleukin IL-1α and IL-1β release by alveolar macrophages and epithelial cells [7–10].

Freshly fractured surfaces are generated by mechanical comminution of quartz-containing materials, which occurs in mining, milling, cutting, sandblasting, and polishing [11, 12]. When quartz is fractured, the newly generated and highly reactive surface radicals may react with environmental water vapor and evolve towards more stable surface silanol moieties (i.e., –SiOH) [6]. In this respect, the nearly free silanols (NFS), which are clustered at a well-defined interatomic distance (i.e., 4–6 Å apart), have been identified on the surface of inflammogenic quartz obtained by fracturing and pyrolytic silica [13], but to a much lesser extent on non-inflammogenic synthetic quartz crystals [14]. The presence of NFS was positively correlated with membranolysis models, including red blood cells and liposomes [4, 5, 14], activation and pro-inflammatory activity of macrophages, and short-term inflammatory responses in rats [14]. No study has yet investigated the effects of NFS on long-term responses to quartz, such as persistent inflammation, fibrosis, lung cancer, or autoimmune responses.

Unresolved inflammation induced by quartz particles leads to fibroblast proliferation [15], transcription of profibrogenic cytokines, i.e., TGF-β and PDGF-AA, increased collagen deposition and fibrosis development [16]. Because of the inflammatory potential of NFS [17], the rationale underlying a possible association between NFS and carcinogenicity is based on the evidence that lung cancer promoted by crystalline silica is driven by a secondary genotoxic mode of action induced by persistent inflammation [18]. Moreover, studies suggest that RCS-induced inflammatory response leads to the dysregulation of the immune system and the onset or exacerbation of autoimmune pathologies. Indeed, chronic exposure to RCS has been associated to the development of autoimmune diseases, including systemic lupus erythematosus, rheumatoid arthritis, systemic sclerosis, and small vessel vasculitis [19, 20]. Pathological features such as oxidative stress, inflammation, and immune system modulation through upregulation of autoantibodies (autoAb) against nuclear antigens, including DNA, could be involved in autoimmune diseases associated with RCS exposure [21].

In this study, we investigated the possible relationship between the occurrence of NFS at quartz surface and the multiple short- and long-term pathogenic outcomes associated with quartz exposure. A set of model quartz particles characterized by rich or poor NFS content were tested in mice. NFS were generated by fracturing synthetic or mineral quartz by milling processes [14]. Quartz particles were administered by oropharyngeal aspiration (o.p.a.) and we assessed the major chronic pathologies resulting from quartz inhalation, including inflammation, fibrosis, cancer, and autoimmunity.

The role of NFS in pulmonary inflammation and fibrosis was investigated in C57BL/6 mice. This model has been previously used to evaluate the fibrotic effect of micro- and nanoparticles, including crystalline silica [22, 23]. A/J mice were used to investigate the promotion of lung cancer by NFS. This strain is susceptible for spontaneous or induced lung cancer, which is characterized by similarities with human lung adenocarcinoma [24, 25]. As quartz is already classified as a carcinogenic agent [3], the co-carcinogenicity approach has been selected, on the basis of quartz secondary genotoxic mechanism [18]. This two-stage initiation-promotion lung tumorigenesis model has been previously used to experimentally elicit lung adenocarcinoma with welding fumes [24, 25] or multi-walled carbon nanotubes [26]. Compared to the classic carcinogenesis test which requires at least 24 months and an important number of animals, the co-carcinogenic model allows to test mechanistic hypotheses on a shorter time and to reduce the number of animals used. Autoimmune responses following quartz exposure were evaluated in 129/Sv mice by assessing the production of anti-dsDNA autoAb, a specific marker of systemic lupus [27]. 129/Sv is an autoimmune-prone strain known to produce autoAb against nuclear components after being exposed to chemical compounds [28]. This strain has previously been tested as responsive to crystalline silica, by developing lung inflammation and fibrosis after intranasal exposure [29].

## Results

### As-grown and fractured quartz particles differ in NFS content, resulting in contrasting membranolytic activities in vitro

**Fig. 1:**
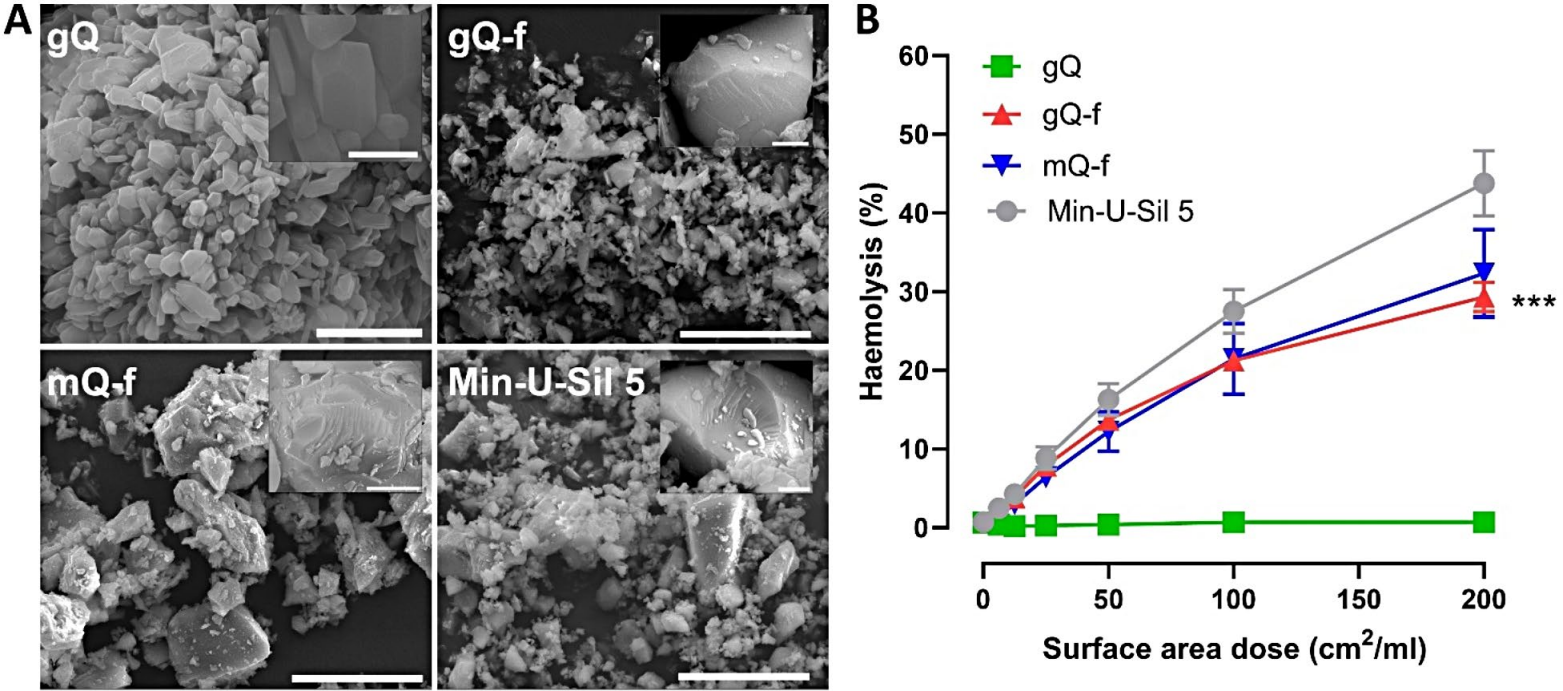
Micromorphology and membranolytic activity of the model quartz particles. (**A**) FE-SEM micrographs of the as-grown synthetic quartz (gQ), fractured as-grown quartz (gQ-f), fractured mineral quartz (mQ-f), and Min-U-Sil 5; relative scale bar, 20 μm, except gQ, 2 μm; scale bar of insets, 1 μm, gQ 400 nm. (**B**) Membranolytic activity (percent haemolysis) at increased concentrations of gQ, gQ-f, mQ-f, and Min-U-Sil 5 (positive reference quartz). Data are mean ± standard error of the mean (SEM) of three independent experiments. *P* values of gQ vs. gQ-f determined by two-way ANOVA followed by Dunnett’s post hoc test (mean effect): ***p < 0.001

To identify a possible relationship between NFS and short- and long-term toxic responses of quartz, a set of model quartz particles characterized by rich or poor NFS content were tested in mice. The physico-chemical features of these particles are summarized in Table 1, and particle morphology assessed by field-emission scanning electron microscopy (FE-SEM) is showed in Fig. 1A. The quartz samples did not differ in other physicochemical features except for NFS, thus allowing the isolation of the contribution of NFS to the toxicological outcome. In particular, the following four quartz samples were considered:

i. one as-grown synthetic quartz (gQ) of respirable size, which was characterized by smooth and well-terminated surfaces, and a very low occurrence of NFS;
ii. one fractured quartz obtained by mechanical comminution of synthetic quartz (gQ-f), which showed conchoidal fractures and irregular surfaces, typical of quartz dusts obtained from mining, and demonstrating abundant NFS;
iii. one fractured quartz obtained by mechanical comminution of macroscopic highly pure quartz crystals of mineral origin (mQ-f), showing occurrence of NFS;
iv. one commercial microcrystalline quartz with a well-documented pathogenic activity (Min-U-Sil 5) [30], which also exhibits NFS.

The model quartz samples were all crystalline and similar in size (the 90% of particles having a diameter below 2 μm), specific surface area (of ca. 4–6 m^2^/g), purity (all quartz samples were pure, except some iron traces in the commercial Min-U-Sil 5), and capacity to generate free radicals (Table 1). Hence, except for quantitative variation in NFS abundance, as-grown and fractured quartz samples showed similar physico-chemical characteristics. We checked both NFS occurrence and membranolytic activity before animal testing, and confirmed the results obtained in a previous study [14]. A negligible amount of NFS (i.e., NFS-poor) was found on gQ, while all fractured samples (gQ-f, mQ-f) and the commercial Min-U-Sil 5, which is also obtained by grinding, showed the presence of the NFS feature (i.e., NFS-rich) (Table 1). The membranolytic activity (i.e., the capacity to lyse red blood cell membranes) of NFS-poor and NFS-rich quartz samples paralleled the occurrence of NFS (Fig. 1B). Overall, the results suggest that NFS-poor gQ and NFS-rich gQ-f, mQ-f, and Min-U-Sil 5 represent convenient particle models to further explore the role of NFS in determining pathogenic responses associated with quartz exposure.

**Table 1:** Physico-chemical features, surface NFS, and membranolytic activity of the model quartz particles

Samples	**Origin**	Physico-chemical features								Membranolytic activity (%)^**g**^ ± SEM
		**SSA** ^**a**^ **(m** ^**2**^ **/g)**	**Morphology** ^**b**^	**Size (µm)** ^**c**^		**Transition metal traces** ^**d**^ **(% wt)**	**Free radical reactivity** ^**e**^		**NFS occurrence** ^**f**^	
				**Diam. ± s.d.**	**D90. ± s.d.**		**HO** ^**•**^	**COO** ^**• –**^		
gQ	as-grown quartz crystals	5.8	Regular, flat surfaces	1.3 ± 2.3	1.4 ± 0.2	0.0	+	absent	low	0.69 ± 0.21
gQ-f	fractured as-grown quartz crystals	3.6	Irregular, heterogeneous surface	1.2 ± 0.6	1.6 ± 0.0	0.0	+	absent	high	21 ± 0.57
mQ-f	fractured mineral quartz	3.8	Irregular, heterogeneous surface	1.2 ± 0.7	1.7 ± 0.0	0.0	absent	absent	high	21 ± 2.6
Min-U-Sil 5	commercial fractured mineral quartz	4.3	Irregular, heterogeneous surface	1.3 ± 0.6	1.8 ± 0.0	Fe 0.3	+	+	high	27 ± 1.3

^a^Brunauer–Emmett–Teller method using Kr; ^b^FE-SEM analysis; ^c^automated flow particle image analysis (*n* = 2–3 measurements); ^d^quantitative scanning electron microscopy – energy dispersive spectroscopy analysis; ^e^spin trapping technique coupled with electron paramagnetic resonance spectroscopy; ^f^diffuse reflectance infrared spectroscopy after H/D exchange; ^g^capacity to lyse the red blood cell membrane (i.e., haemolysis) for the quartz concentration of 100 cm^2^/ml

### NFS-rich quartz induces acute and persistent pulmonary inflammation in mice

**Fig. 2:**
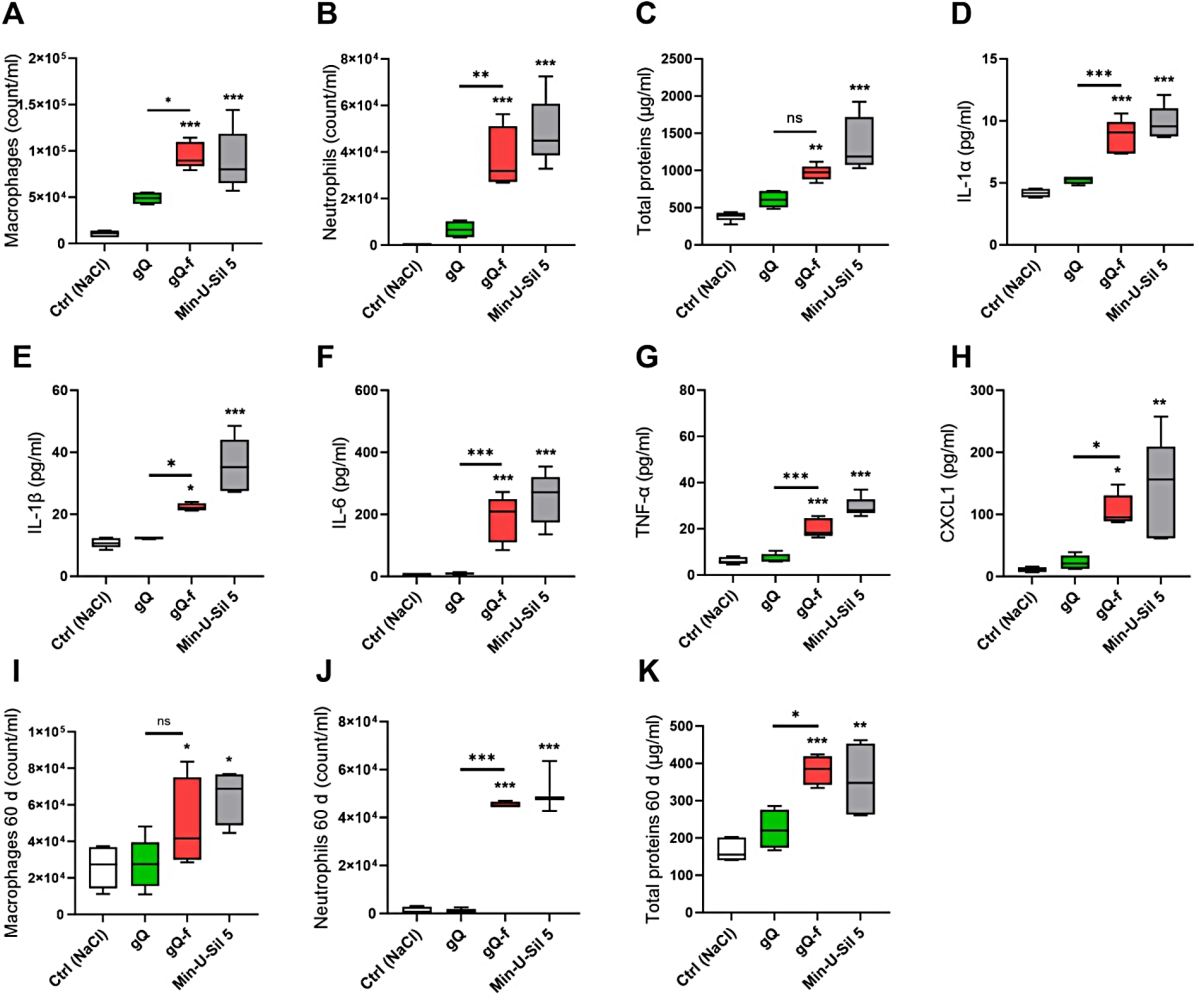
Acute and persistent pulmonary inflammation in mice is associated with exposure to NFS-rich quartz. C57BL/6 mice were exposed via oropharyngeal aspiration to physiological saline (Ctrl), or 2 mg of NFS-poor (gQ) or NFS-rich (gQ-f and Min-U-Sil) quartz particles. Mice were sacrificed after 3 and 60 days from particle administration. Inflammation was investigated in the BALF. Acute inflammation (3 days): (**A**) macrophage count, (**B**) neutrophil count, (**C**) total proteins, (**D**) IL-1α, (**E**) IL-1β, (**F**) IL-6, (**G**) TNF-α, and (**H**) CXCL1. Persistent inflammation (60 days): (**I**) macrophage count, (**J**) neutrophil count, (**K**) total proteins. *p < 0.05, **p < 0.01, and ***p < 0.001 relative to Ctrl mice or gQ vs. gQ-f-exposed mice. Data were analysed with ANOVA and Tukey’s multiple comparisons test, *n* = 5 per group, and are presented as box and whiskers plots with median, min and max values, and 25th to 75th percentile

The acute (Fig. 2A-H) and persistent (Fig. 2I-K) lung inflammation was assessed in C57BL/6 mice treated with 2 mg [23, 31] to gQ, gQ-f, and Min-U-Sil 5 after 3 and 60 days, respectively. The acute inflammatory response to the NFS-poor gQ was minor, overall showing levels comparable to the control group, i.e., physiological saline (Fig. 2A-H). No statistically significant increase in macrophage or neutrophil accumulation was observed in the bronchoalveolar lavage fluid (BALF) of gQ-exposed mice (Fig. 2A-B). The total protein concentration in BAL, which is a measure of the alveolar-capillary barrier permeability and inflammation (Fig. 2C), and the secretion of cytokines (Fig. 2G-J) and chemokines (Fig. 2K) were not increased after gQ. In contrast, the NFS-rich gQ-f induced massive recruitment of macrophages and neutrophils (Fig. 2A-B). The alveolar permeability upon gQ-f treatment was not statistically different from gQ (Fig. 2C), whereas BALF levels of the pro-inflammatory cytokines IL-1α (Fig. 2D), IL-1β (Fig. 2E), IL-6 (Fig. 2F), TNF-α (Fig. 2G) and CXCL1 (Fig. 2H) significantly increased compared to gQ. The acute inflammatory responses after gQ-f exposure were comparable to those of quartz Min-U-Sil 5. Lung inflammation was persistent (i.e., macrophage and neutrophil recruitment, Fig. 2I-J, and alveolar capillary permeability, Fig. 2K) 60 days after NFS-rich quartz (i.e., gQ-f and Min-U-Sil 5) administration. Saline and gQ instillation did not induce long-term inflammatory responses (Fig. 2I-K). These data indicate that NFS-rich quartz led to acute and chronic pulmonary inflammation, while NFS-poor quartz only induced a minor inflammatory effect, suggesting the crucial role of NFS in the development of lung inflammation upon quartz exposure.

### NFS-rich quartz induces long-term lung fibrotic responses

**Fig. 3:**
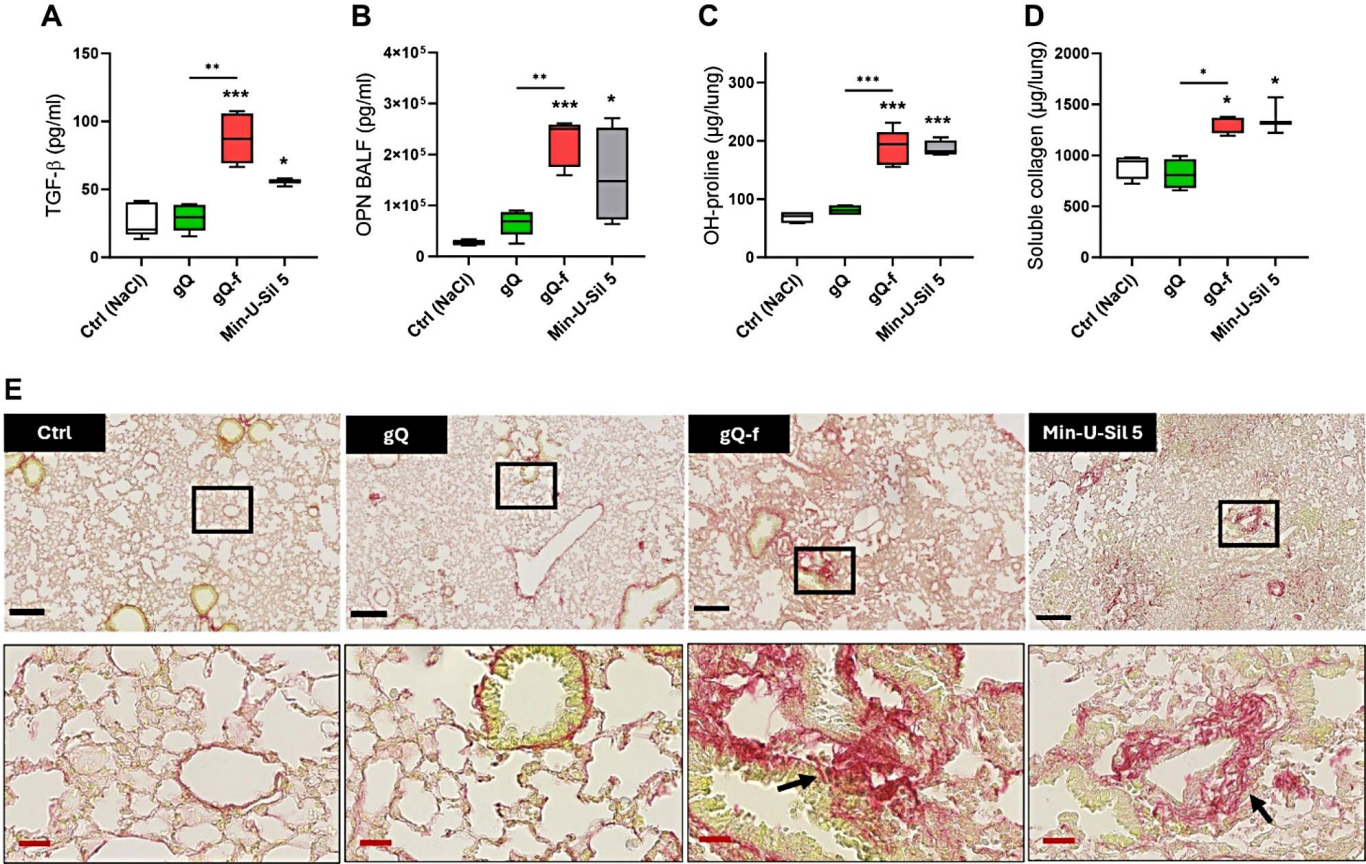
Lung fibrotic response in mice is associated with exposure to NFS-rich quartz. C57BL/6 mice were exposed via oropharyngeal aspiration to physiological saline (Ctrl), or 2 mg of NFS-poor (gQ) or NFS-rich (gQ-f and Min-U-Sil 5) quartz particles. After 60 days, fibrosis biomarkers were investigated in the BALF, i.e., (**A**) TGF-β, and (**B**) osteopontin, or in homogenized lungs, i.e., (**C**) hydroxyproline, and (**D**) soluble collagen. (**E**) Histology was assessed on lung sections stained with Picrosirius Red, highlighting type I collagen accumulation. Black bar: 100 μm, red bar: 25 μm. Black boxes indicate the area that has been magnified, black arrows indicate collagen fibers. *p < 0.05, **p < 0.01, and ***p < 0.001 relative to Ctrl mice or gQ vs. gQ-f-exposed mice. Data were analysed with ANOVA and Tukey’s multiple comparisons test, *n* = 5 per group, and are presented as box and whiskers plots with median, min and max values, and 25th to 75th percentile

To evaluate the role of NFS in driving fibrotic pulmonary responses, C57BL/6 mice were exposed by o.p.a. to 2 mg of quartz particles (i.e., gQ, gQ-f, and Min-U-Sil 5). Sixty days after the exposure, mice were sacrificed, and the BALF levels of TGF-β and osteopontin (OPN), two key regulators of fibrosis [32, 33], were monitored. Lung collagen accumulation was assessed by measuring total lung hydroxyproline content, soluble collagen (Sircol assay), and histology via Picrosirius red staining. NFS-poor gQ did not induce profibrotic responses (Fig. 3). TGF-β (Fig. 3A), OPN (Fig. 3B), and collagen accumulation (Fig. 3C-D) were not increased following gQ administration in comparison to controls. In contrast, profibrotic responses were induced by NFS-rich particles. Indeed, gQ-f induced significant increase in both TGF-β and OPN levels when compared to gQ (Fig. 3A-B). Hydroxyproline quantification (Fig. 3C) and measurement of the soluble collagen (Fig. 3D) also increased, indicating pulmonary collagen accumulation in mice exposed to gQ-f. Similarly, profibrotic responses were observed after Min-U-Sil 5 (Fig. 3A-D). Histological analysis of lung sections (Fig. 3E) revealed inflammatory areas and excessive collagen deposition (red stained) only upon exposure to NFS-rich (gQ-f, Min-U-Sil 5), but not NFS-poor quartz (gQ). Our data thus showed that NFS-rich quartz, but not NFS-poor quartz, induces pulmonary collagen accumulation and subsequent fibrosis. We concluded that NFS could be a key molecular determinant of quartz-induced fibrogenesis.

### NFS-rich quartz promotes lung adenocarcinoma

**Fig. 4:**
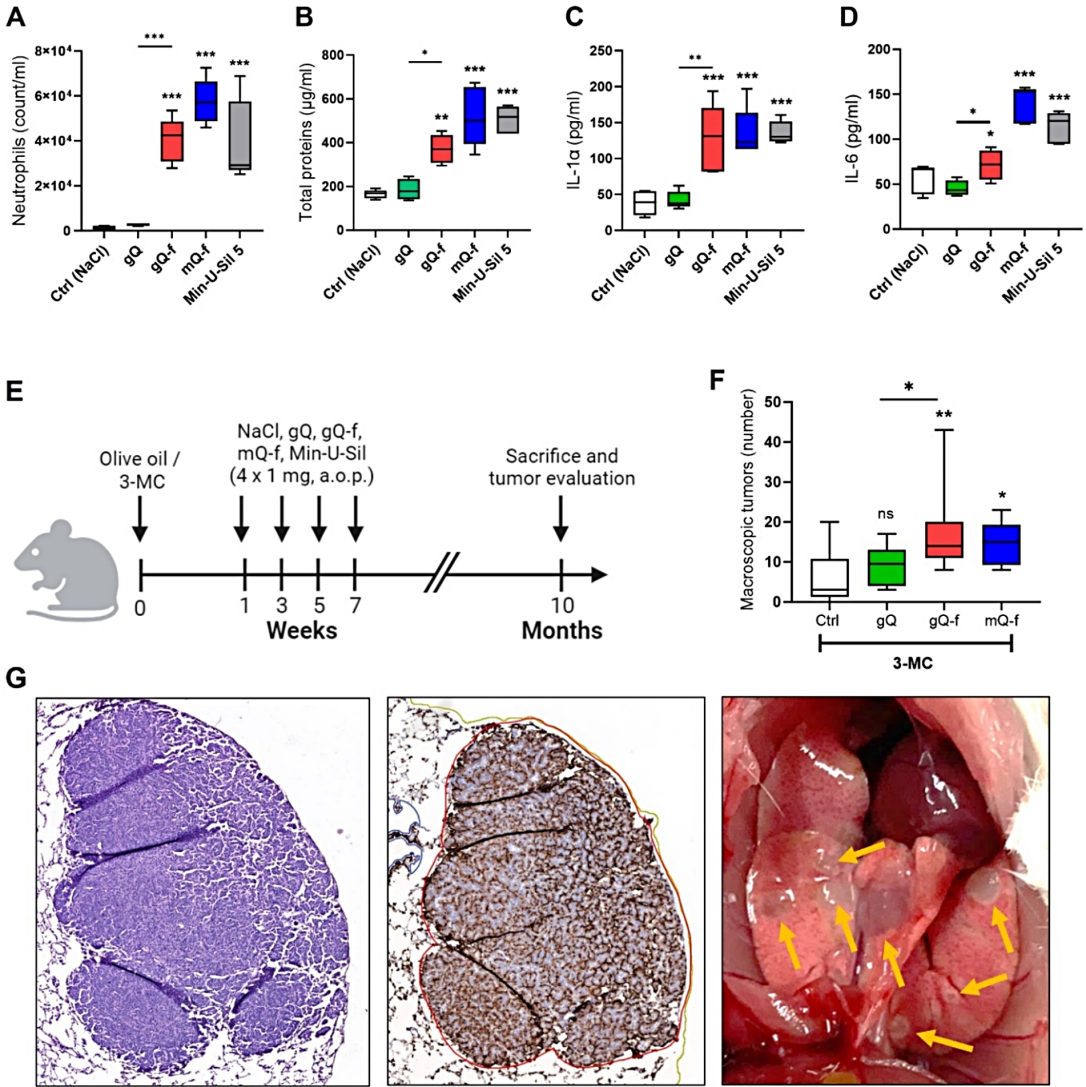
Promotion of lung cancer by quartz is associated with exposure to NFS-rich quartz. **(A-D)** Acute proinflammatory responses were measured in the BALF of A/J mice 3 days after the exposure to NFS-poor (gQ) or NFS-rich (gQ-f, mQ-f, Min-U-Sil 5) quartz particles (2 mg/mouse, o.p.a., *n* = 5 per group). Controls (Ctrl) were treated with physiological saline. (**A**) Neutrophil count, (**B**) total proteins, (**C**) IL-1α, and (**D**) IL-6 dosage in the BALF. **(E-G)** Macroscopic quantification of lung tumour by counting nodules on the surface of the lung of A/J mice pre-treated with 3-MC (1 week) and exposed (1 mg x 4) to gQ, gQ-f, or mQ-f, at 10 months after exposure. (**E**) Lung exposure strategy, (**F**) lung tumour multiplicity upon gross examination at necropsy. (**G**) Representative images of adenocarcinoma in the lungs of animals exposed to mQ-f quartz: haematoxylin staining (left), MUC1 immunostaining (centre), lung tissue with tumour burden (right). *p < 0.05, **p < 0.01, and ***p < 0.001 relative to Ctrl mice or gQ vs. gQ-f-exposed mice. Data were analysed with ANOVA and Tukey’s multiple comparisons test and are presented as box and whiskers plots with median, min and max values, and 25th to 75th percentile

The involvement of NFS in quartz carcinogenicity was addressed in A/J mice. We first assessed the susceptibility of this mouse strain to NFS by investigating proinflammatory responses 3 days after the exposure to NFS-poor (gQ) or -rich (gQ-f, Min-U-Sil 5) quartz particles (2 mg/mouse). As observed in C57BL/6 mice, gQ quartz treatment did not induce acute inflammation in A/J mice, while gQ-f exposure induced a neutrophil recruitment (Fig. 4A), increased alveolar permeability (Fig. 4B) and increased BALF levels of IL-1α and IL-6 (Fig. 4C-D). These results confirmed the sensitivity of A/J mice to the NFS-associated toxic potential.

For setting up the two-stage lung tumour bioassay, A/J mice were pre-treated (initiation stage) with 3-methylcholantrene (3-MC) (Fig. 4E). 3-MC is a direct genotoxic substance, which directly interacts with DNA via covalent bonds and induces mutations. Later, 3-MC pre-treated animals were exposed to quartz (1 mg) every two weeks for a total of four times, to mimic repeated particle exposure (promotion stage) [25] (Fig. 4B). Ten months after 3-MC injection, mice were sacrificed for necroscopy and histological analysis of lung tissues. Three mice exposed to NFS-rich Min-U-Sil 5 died prematurely before the experiment concluded, indicating a potential onset of massive lung adenocarcinoma. The pronounced tumour progression was corroborated by immunohistochemistry analysis (see below) in six additional mice exposed to NFS-rich Min-U-Sil 5, which were sacrificed at interim points. Due to the extensive cancer development observed with Min-U-Sil 5, we selected mQ-f, an NFS-rich quartz dust, as an alternative silica sample [14]. We demonstrated its ability to induce comparable inflammation in A/J mice with respect to Min-U-Sil 5 (Fig. 4A-D, blue columns), without affecting the survival rate of treated mice.

Tumours were macroscopically counted after co-treatment of A/J mice with 3-MC and gQ, gQ-f and mQ-f (Fig. 4F). After 10 months, no tumour exacerbation was observed in mice exposed to NFS-poor gQ quartz when compared to the control group. On the contrary, tumour number was significantly increased in gQ-f-exposed mice. In addition, higher tumour multiplicity (1.8-fold) was observed in NFS-rich gQ-f-treated mice with respect to gQ (Fig. 4F). Augmented tumour multiplicity was also detected in animals exposed to NFS-rich mQ-f quartz (Fig. 4F). Haematoxylin and mucine-1 (MUC1) staining on tissue sections collected from mice exposed to mQ-f quartz confirmed that the observed pulmonary lesions were adenocarcinomas (Fig. 4G). Gross lung morphology showed that the size of tumours was between 0.5 mm and 3 mm, and tumours appeared white in colour and well delimited (Fig. 4G). These results support a role of NFS in promoting the carcinogenic potential of fractured quartz particles.

### NFS-rich quartz induces systemic autoimmune responses

The onset of autoimmunity hallmarks, including the production of autoAb, is recognized in mice exposed to crystalline silica dusts [21, 34, 35]. Here, we assessed the impact of NFS in promoting silica-induced autoimmune responses in 129/Sv mice, an inbred strain prone to autoAb production [28] and known to develop lung inflammation and fibrosis when exposed to quartz particles [29]. Total serum IgG and anti-dsDNA autoAb, which are markers of autoimmunity, were quantified 4 months after gQ, gQ-f and Min-U-Sil 5 exposure (2.5 mg per mouse). This specific dose was chosen because it is able to trigger inflammatory and fibrotic responses in 129/Sv mice [36].

**Fig. 5:**
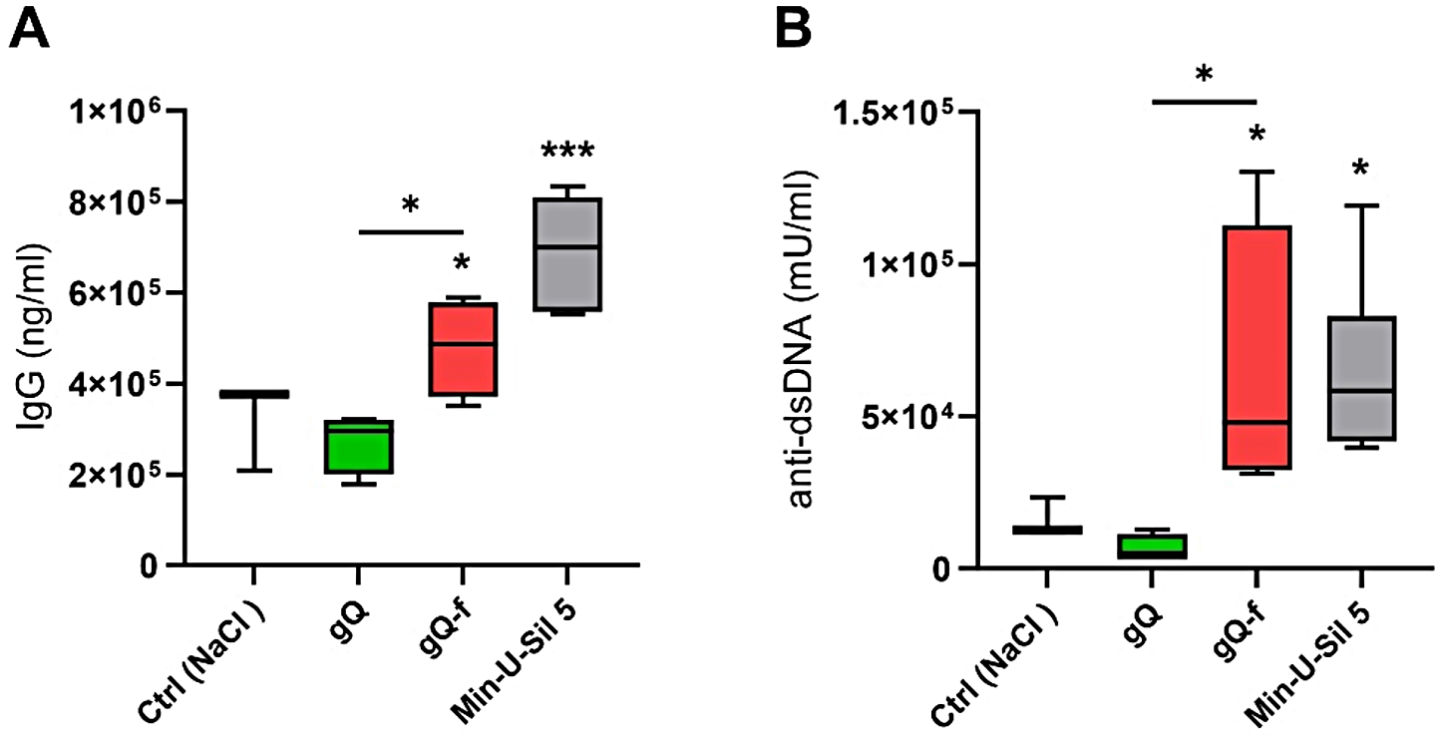
Production of autoantibodies in 129/Sv mice is triggered by NFS-rich quartz. Male 129/Sv mice were exposed via o.p.a. to physiological saline (Ctrl), or 2.5 mg of NFS-poor (gQ) or NFS-rich (gQ-f, Min-U-Sil 5) quartz particles. The onset of autoimmune response was evaluated by quantifying serum levels of IgG (**A**) and anti-dsDNA autoantibodies (**B**). *p < 0.05 and ***p < 0.001 relative to Ctrl mice or gQ vs. gQ-f-exposed mice. Data were analysed with ANOVA and Tukey’s multiple comparisons test, *n* = 8 per group, and are presented as box and whiskers plots with median, min and max values, and 25th to 75th percentile

In gQ-exposed mice, the baseline serum levels of IgG (Fig. 5A) and anti-dsDNA autoAb (Fig. 5B) were not affected. On the contrary, NFS-rich gQ-f particles significantly elevated both IgG and anti-dsDNA autoAb levels (1.6-fold and 10-fold, respectively) compared to the control group and gQ. A significant increase of autoAb production was also observed following Min-U-Sil 5 quartz exposure. This result suggests that NFS occurrence is related to the potential of quartz particles to induce autoimmune dysfunctions.

## Discussion

Our study is the first to highlight that some of the chronic responses induced by the inhalation of quartz share a common biomolecular origin, represented by surface nearly-free silanols (NFS). The NFS represent a specific population of weakly interacting silanols generated through fracturing of quartz crystals and have been recently pointed out as the molecular structures initiating toxic activity of silica particles [14]. During the process of surface reconstruction, occurring after fracturing, highly reactive surface features (i.e., surface radicals) recombine to generate more stable species [6, 37]. Among these newly formed species, the NFS are more energetically favoured for interacting with membrane phospholipids, playing a key role in triggering silica toxicity [5, 14]. We previously observed that NFS-rich, but not NFS-poor, silica particles induced the release of the pro-inflammatory cytokine IL-1β from macrophages [14]. Our present data offer a more comprehensive scenario about the key role played by NFS in driving the pathogenic activity of quartz. While NFS-poor quartz was essentially non-toxic in mice, NFS-rich quartz induced strong, short- and long-term toxic responses. Indeed, exposure to NFS-rich quartz particles (i.e., gQ-f, mQ-f, and Min-U-Sil 5) resulted in the persistent recruitment of inflammatory cells, cytokine and chemokine release, collagen deposition, fibrosis development and tumour multiplicity increase in the lungs. Systemic and autoimmune responses (i.e., serum IgG and anti-dsDNA autoAb) were also related to NFS abundance. These responses were significantly less pronounced in mice exposed to NFS-poor gQ quartz. The pathological features of the local and systemic responses that we observed in mice treated with NFS-rich quartz are in line with the knowledge on quartz toxicity, which includes pulmonary infiltration of phagocytic cells and neutrophils [38, 39], cytokine (i.e., IL-1α, IL-1β, IL-6, TNF-α) and chemokine (i.e., CXCL1) secretion, increased collagen deposition [40–43], tumour growth [44], and autoimmune disorders [21, 45, 46]. These features were either absent or significantly less pronounced in mice exposed to NFS-poor quartz.

Mechanistically, the capacity of NFS to destabilize phospholipid membranes through a specific molecular recognition mechanism has been recently demonstrated [5]. Furthermore, NFS-rich, but not NFS-poor quartz, triggered lysosomal stress and destabilization following particle internalization by macrophages [7]. Lysosomal membrane permeabilization (LMP) is a key signal inducing the assembly of the NLRP3 inflammasome [47, 48], which in turn mediates the activation of inflammatory caspase-1, resulting in the maturation and release of IL-1α and IL-1β cytokines [49–51]. We previously observed that the release of both IL-1α and IL-1β occurred in the presence of NFS-rich quartz, but not with NFS-poor quartz particles [9, 14].

It has been shown that quartz-activated macrophages induce the persistent recruitment of neutrophils via release of IL-1 cytokines and CXCL chemokines, which are commonly overexpressed during inflammatory responses [31, 52]. Notably, we observed neutrophil influx in all the mice strains exposed to NFS-rich quartz. Moreover, our data show that neutrophilic inflammation persisted and progressed into fibrosis even after cessation of exposure to NFS-rich quartz, but not to NFS-poor quartz. Both NFS-rich and NFS-poor quartz are crystalline in structure, thus this finding adds additional information about the prominent role of crystal surface, compared to crystalline structure, in triggering persistent, progressive responses after cessation of silica exposure, as recently highlighted for amorphous vs. crystalline silica [43]. Several studies suggest the relevance of persistent neutrophil influx in the progression of pulmonary fibrosis [53]. Neutrophil extracellular traps (NETs), which are produced by activated neutrophils in response to crystalline silica, have been shown to promote tissue damage and pulmonary fibrosis [54]. These NET components may also play a role in the initiation and progression of autoimmune responses [55]. Persistent neutrophilic inflammation has also been linked to quartz genotoxicity and carcinogenicity [56]. Oxidant factors generated by proinflammatory neutrophils promote DNA injury and amplify genome instability and mutations in epithelial cells [18, 57, 58].

In this study, we did not assess the impact of NFS on cell oxidative stress that can boost inflammatory, fibrogenic, and genotoxic effects initiated by quartz particles [59, 60]. Following LMP, quartz particles released in the cytosol can also impact the intracellular respiratory chain inducing mitochondrial dysfunction and depolarization of the mitochondrial membrane, ultimately leading to ROS release and inflammasome assembly [61]. We may speculate that in the presence of NFS-rich quartz, LMP and oxidative stress occur, whereas with NFS-poor quartz, neither LMP nor oxidative stress take place. In any case, a contribution of particle-induced ROS (Table 1) in triggering membranolysis or early inflammatory responses might be excluded, as previously observed [14]. Further research could explore the oxidative mechanisms possibly related to NFS and broaden the range of the examined silica types to confirm the applicability of the NFS model to other crystalline silica polymorphs and amorphous silica.

## Conclusions

Our results unveil novel insights on the critical role of the surface NFS in determining short- and long-term toxic responses associated with quartz exposure. NFS, which occur on fractured quartz particles, induced pulmonary inflammation, fibrosis, cancer, and autoimmunity, while very low or no activity was observed for NFS-poor quartz. Persistent neutrophilic inflammation emerges as a key event in the promotion of local (fibrosis, cancer) and systemic (autoimmunity) pathogenicity induced by NFS-rich quartz. This study supports surface NFS as an effective predictor of the toxic activity of silica-based materials.

## Materials and methods

### Quartz sample preparation and physico-chemical characterisation

As-grown quartz crystals (gQ) were obtained in respirable size by hydrothermal synthesis, as previously described [14, 62]. To obtain the fractured as-grown quartz (gQ-f), 500 mg of the larger fraction (> 30 μm) of the sieved gQ was ground in a mixer mill (Retsch MM200) at 27 Hz for 6 h [14]. Agate jars and two agate balls of 6 mm diameter per jar were used to avoid contamination. The parameters were selected to attain bulk characteristics similar to those of the reference quartz dust (Min-U-Sil-5). The mineral fractured quartz (mQ-f) was obtained by ball milling of millimetric very pure quartz crystals from Madagascar, as indicated elsewhere [14]. A systematic analysis of the particle size distribution was previously conduced to select the grinding parameters [14]. The commercial microcrystalline quartz (Min-U-Sil 5, U.S. Silica Co.) was used as a positive reference particle because of its well-documented toxic activity [30]. All quartz particles were fully characterized for particle crystallinity, size and morphology, elemental analysis, surface chemistry, capacity to release free radicals by using x-ray diffraction, FE-SEM, automated flow particle image analysis, energy dispersive x-ray spectroscopy, Brunauer–Emmett–Teller method, electrophoretic light scattering, and spin trapping technique coupled with electron paramagnetic resonance spectroscopy, in previous studies [5, 7, 14]. For NFS determination, the surface silanols (SiOH) were converted in the SiOD form by isotopic exchange with D_2_O and diffuse reflectance infrared spectroscopy was applied, as previously described in detail [14, 63]. The membranolytic activity of the samples was assessed by their ability to lyse the red blood cells (i.e., haemolysis test) at increasing particle dose, considering the exposed surface area [14]. The measure of the NFS and membranolytic activity were monitored before animal testing.

For administration to mice, particles were sterilized at 200 °C for 2 h, and suspensions were prepared in physiological saline (NaCl 0.9%) at a concentration of 20 mg/ml. Discrete sonication in an ultrasonic bath (USC100T; VWR) was used to disperse particle suspensions, which were serially diluted in saline when required.

### Pulmonary inflammation and fibrosis

Eight-week-old female C57BL/6JRj mice were purchased from Janvier Labs (Saint Berthevin, France). Animals were housed in a SPF-like animal facility in positive-pressure air-conditioned units (25 °C, 50% relative humidity) on a 12 h light/dark cycle, with ad libitum access to water and food. Mice were randomly allocated to experimental groups (*n* = 5/group), and data collection and analysis were performed blind. After anaesthesia with a mix of Rompun 0.2 mg/mouse (Bayer), and Nimatek, 1 mg/mouse (Eurovet), via intraperitoneal injection, the animals were treated with the particle suspensions via oropharyngeal aspiration (o.p.a., 50 µl/mouse). Control animals were treated with 0.9% NaCl (50 µl/mouse). The dose of silica was selected on the basis of previous results showing efficient lung delivery and pro-inflammatory/profibrotic responses in mice, overcoming long-term pulmonary clearance mechanisms [22, 31, 64]. Animals were euthanized 3 days (acute inflammation) or 60 days (long-term inflammation and fibrosis) after particle administration with an intraperitoneal injection (60 mg/ml, 200 µl) of sodium pentobarbital (Certa). These time intervals were selected according to previous studies [36, 65].

### Bronchoalveolar lavage and lung sampling

Broncho-alveolar lavage (BAL) was performed by cannulating the trachea and infusing the lungs with 1 ml 0.9% NaCl. The BAL was then centrifuged at 250 *g*, 4 °C, 10 min (Centrifuge 5804R, Eppendorf). The cell-free supernatant (BALF) was used for biochemical measurements and the cellular fraction was resuspended in phosphate buffer saline (PBS) for cell counting. Total BALF cells were counted in Turch (crystal violet 1%, acetic acid 3%) and pelleted onto glass slides by cytocentrifugation for differentiation by light optical microscopy by Diff-Quick staining (Polysciences). Two hundred cells were considered.

Whole lungs were perfused with 0.9% NaCl and excised. Left lobes were placed in a 3.6% paraformaldehyde (Sigma-Aldrich) neutral buffered solution and embedded in paraffine for histological analysis. The right lobes were frozen in liquid nitrogen and homogenized in 2 ml of PBS on ice with an Ultra-Turrax T25 (Janke and Kunkel) and stored at -80 °C.

### Quantification of lung inflammatory, cytotoxic, and fibrotic markers

The following enzyme-linked immunosorbent assays (ELISA) were performed on the BALF collected from mice, according to manufacturers’ instructions (DuoSet ELISA, R&D Systems): IL-1α (DY400, limit of detection (LOD): 7.8 pg/ml), IL-1β (DY401, LOD: 7.8 pg/ml), IL-6 (DY406, LOD: 7.8 pg/ml), CXCL1/KC (DY453, LOD: 7.8 pg/ml), TNF-α (DY410, LOD: 15.6 pg/ml), TGF-β (DY1679, LOD: 15.6 pg/ml), and OPN (DY441, LOD: 7.8 pg/ml). The total proteins present in the BALF were measured by an absorbance test according to the supplier’s instructions (Pierce BCA Protein Assay Kit, Thermo Scientific).

Collagen deposition was estimated on the supernatant of hydrolysed lung homogenates (6 N HCl, 108 °C during 24 h) obtained by centrifugation (5 min at 2800 *g*) using the Sircol-soluble collagen assay (BioColor) according to the manufacturer’s protocol, or by measuring the hydroxyproline content in lung homogenates as described previously [66].

### Histology

Paraffine-embedded sections (thickness = 5 μm) were stained with Picrosirius Red (fibrosis analysis, type I and III collagen staining). The sections were then scanned with a Leica SCN400 slide scanner (Leica) and examined with Tissue Image Analysis 2.0 (Leica Biosystems).

### Co-carcinogenicity test

Six-week-old male A/J mice (Jackson Laboratory, Bar Harbor, U.S) were housed as previously explained. After acclimation (more than one week), mice were firstly tested for their capacity to develop quartz-induced proinflammatory reactions in the lungs. Animals were treated (50 µl/mouse) with 2 mg of NFS-poor (gQ) or NFS-rich (gQ-f, mQ-f, Min-U-Sil 5) quartz particles. Controls animals were treated with 0.9% NaCl (50 µl/mouse). 3 days after the exposure, animals were euthanized and the BALF collected and analysed for neutrophil number, total protein content, IL-1α, and IL-6 levels. For carcinogenesis evaluation, mice were randomly allocated to experimental groups (*n* = 10–12/group) and treated via intraperitoneal injection (i.p.) with 10 µg/g of body weight with a solution of 3-methylcolantrene (3-MC, Sigma, 300 µl/mouse) dissolved in ethyl acetate (5%) and diluted in olive oil. 3-MC was previously used as a chemical initiator in co-carcinogenesis studies carried out in A/J mice [24, 25]. One week after, mice were exposed to 1 mg of particles in 50 µl of saline via o.p.a. for 4 times (4 × 1 mg/mouse). Each particle dose was administered with an interval of 2 weeks. Ten months after exposure to 3-MC, mice were euthanized with an overdose of sodium pentobarbital (intraperitoneal injection, 60 mg/ml, 200 µl/mouse). This time point was chosen to uncouple the effects of 3-MC and silica, in accordance with the study of Freire and co-workers [67]. The rib cage was opened, the vena cava cut, and images of the lungs were taken. One ml of 3.6% paraformaldehyde was slowly injected into the lungs via the trachea. After excision, the lungs were photographed double-sided for superficial macroscopic analysis of the tumours and stored in a 3.6% paraformaldehyde neutral buffered solution (Sigma-Aldrich) for at least 24 h. The lungs were embedded in paraffin for histological analysis.

### Detection of MUC1 in mouse lung by immunohistochemistry

Lung sections were deparaffinized in toluene (3 × 5 min) and isopropanol (3 × 5 min), and then rehydrated in gradient methanol. To improve antigen retrieval, the samples were placed in methanol with 3% H_2_O_2_ to block endogenous peroxidases (20 min) and then treated with 0.1 M sodium citrate buffer (pH 5.7, 100 °C). After washes in a 0.05% Tris-Buffered Saline (TBS)/Triton solution (0.05%), sections were blocked for the non-specific binding with 5% bovine serum albumin (BSA) diluted in the TBS/Triton solution. Samples were then incubated overnight with the primary Ab (rabbit anti-MUC1, dilution 1:500, cat. # MA5-35250; Invitrogen, Waltham, MA, USA) at 4 °C. After incubation (45 min, 25 °C) with secondary peroxidase-labelled anti-rabbit Ab (1:150, cat. # K3468; Dako, Glostrup, Denmark), reaction was carried out with 3–3’-diaminobencidine and 0.03% H_2_O_2_ in TBS. Visualization of immunostaining was performed using diaminobenzidine (Sigma, St. Louis, MO, USA). Sections were finally counterstained with haematoxylin (Sigma), dehydrated and mounted using Tissue-Tek film (Sakura, Japan). Samples were evaluated under light microscope. Histological analysis was independently conducted by two experts.

### Autoimmunity

Five-month-old male 129/Sv mice (Janvier Labs, Saint Berthevin, France) were housed as previously described. Mice were exposed to sterile particle suspensions (2.5 mg/mouse) via o.p.a. This dose of crystalline silica is known to induce immune responses in the mouse strain used [36]. Furthermore, a single administration of this dose of Min-U-Sil 5 quartz triggered autoimmunity in previous studies carried out in lupus-prone mice [68]. Mice were euthanized 4 months after particle administration with an intraperitoneal injection of sodium pentobarbital (60 mg/ml, 200 µl). This time interval agrees with previous studies investigating silica-associated autoimmune activity [21, 46].

### Serum immunoglobulins and autoantibodies

Blood samples (1 ml) were collected via cardiac puncture from the left ventricle of anesthetized 129/Sv mice. Serum was isolated by centrifugation and then frozen at − 80 °C until being analysed for the concentrations of IgG and anti-dsDNA autoantibodies via ELISA (Invitrogen ThermoFischer Scientific LOD: 1.6 ng/ml, and ELK Biotechnology LOD: 0.16 ng/ml, respectively).

### Statistical analysis

Statistical parameters, including the number of independent experiments, the number of biological replicates per experiment, and statistical significance, are reported in the figures and figure legends. Normally distributed data were analysed by one-way ANOVA followed by Tukey’s post hoc test. In all tests, a 95% confidence interval was used, for which differences with *P* < 0.05 were considered statistically significant. Data are presented as box plots and whiskers plots with median, min and max values, and 25th to 75th percentile. Statistical analysis was performed with the GraphPad Prism 10 software (GraphPad Software, La Jolla, CA, USA).

## Data Availability

The authors declare that the data supporting the findings of this study are available within the manuscript or its supplementary information files.
